# Structural Analysis of a Novel Class of R–M Controller Proteins: C.Csp231I from *Citrobacter* sp. RFL231

**DOI:** 10.1016/j.jmb.2011.03.033

**Published:** 2011-06-03

**Authors:** J.E. McGeehan, S.D. Streeter, S.-J. Thresh, James E N Taylor, M.B. Shevtsov, G.G. Kneale

**Affiliations:** Biophysics Laboratories, Institute of Biomedical and Biomolecular Sciences, School of Biological Sciences, University of Portsmouth, Portsmouth PO1 2DY, UK

**Keywords:** restriction–modification, helix–turn–helix, X-ray crystallography, small-angle X-ray scattering, analytical ultracentrifugation

## Abstract

Controller proteins play a key role in the temporal regulation of gene expression in bacterial restriction–modification (R–M) systems and are important mediators of horizontal gene transfer. They form the basis of a highly cooperative, concentration-dependent genetic switch involved in both activation and repression of R–M genes. Here we present biophysical, biochemical, and high-resolution structural analysis of a novel class of controller proteins, exemplified by C.Csp231I. In contrast to all previously solved C-protein structures, each protein subunit has two extra helices at the C-terminus, which play a large part in maintaining the dimer interface. The DNA binding site of the protein is also novel, having largely AAAA tracts between the palindromic recognition half-sites, suggesting tight bending of the DNA. The protein structure shows an unusual positively charged surface that could form the basis for wrapping the DNA completely around the C-protein dimer.

## Graphical Abstract

**Figure:**
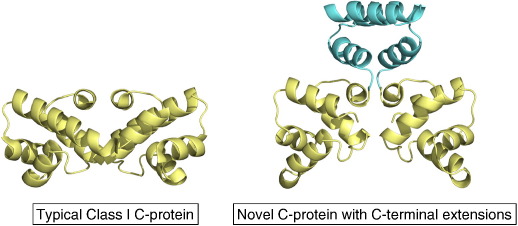

**Research Highlights:**

► A novel R–M controller protein has been crystallised. ► DNA binding studies demonstrate complex formation with its promoter. ► The crystal structure of C.Csp231I reveals an extended C-terminal domain. ► Analytical ultracentrifugation and small-angle X-ray scattering studies confirm that this extended region is folded in solution. ► An unusual surface charge distribution is discussed in relation to DNA binding.

## Introduction

Bacterial restriction–modification (R–M) systems employ a range of mechanisms for the temporal regulation of methylase and restriction endonuclease.^1^ In many type II systems, this is achieved at the transcriptional level by the action of a small helix–turn–helix controller protein, or C-protein.^2^, ^3^, ^4^, ^5^, ^6^, ^7^ Following the transfer of an R–M system into a naive host, the action of the restriction endonuclease must be delayed until the host DNA is protected from cleavage following specific methylation by the methylase. Loss of this temporal control has been shown to lead to degradation of the host genome and cell death *in vivo*,^8^ and has been modelled *in silico*.^9^ Since the presence of R–M systems in bacterial populations is directly related to the horizontal transfer of genetic information,^10^ including antibiotic resistance,^11^, ^12^, ^13^ it is of particular interest to understand the structure and mechanism of such control systems.

Recent studies have identified over 290 potential C-proteins in the DNA sequence database.^14^ However, only a small proportion of these genes have been shown to encode functional proteins. C-proteins have been divided into several classes based on motifs in their (predicted) DNA recognition sites and/or amino acid sequences.^14^, ^15^ X-ray crystallographic and functional information now exists for the AhdI,^16^, ^17^, ^18^, ^19^, ^20^ BclI^21^ and Esp1396I^22^, ^23^, ^24^ systems, while other systems such as PvuII, although extensively studied *in vitro* and *in vivo*,^7^, ^8^, ^15^ currently lack any structural data. Together, these studies have revealed a highly cooperative, concentration-dependent genetic switch that allows the fine temporal control necessary to establish and to maintain an active R–M system in bacteria.

Almost all of the C-proteins that have been studied to date have two operators, each consisting of quasi-palindromic sequences usually of the form GACTtatAGTC, separated by a central and highly conserved GT. In addition, the symmetric sequence TG…CA is frequently found outside of the central sequence elements, with the entire DNA binding site being around 35 bp long; indeed, structural and functional analysis shows that these outer bases play a vital role in protein–DNA recognition.^23^, ^24^ To date, all structural studies have been confined to this class of C-protein.

**Fig. 1:**
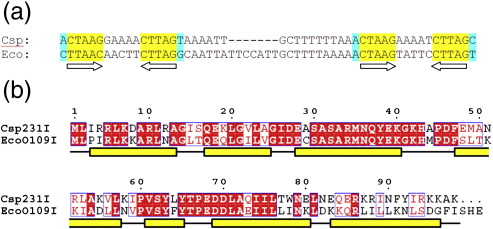
Comparison of C.Csp231I and C.EcoO109 amino acid sequences and putative binding sites. (a) The binding region of C.Csp231I is shown with the inverted repeats (arrows) highlighted in yellow; these form four perfectly palindromic half-sites in C.Csp231I. An extra base (A/T; cyan) might also be recognised, as it is present in three of the four half-sites in C.Csp231I. Related palindromic sites can be found upstream of the C.EcoO109I gene, although there are an additional  7 bp in the spacer region compared to C.Esp231I. (b) An alignment of C.Csp231I and EcoO109I is shown, with identical amino acids highlighted in red boxes and with similar residues shown in red text. The positions of the seven α-helices from the C.Csp231I crystal structure are shown as yellow boxes under the sequence.

However, Sorokin *et al.* recently identified additional classes of C-proteins based on a bioinformatic analysis of their DNA recognition sites.^14^ We were particularly interested in the class exemplified by the R–M systems EcoO1091I and Csp231I, since their recognition sequences (classified by Sorokin *et al.* as motif 8) have unusual features.^25^, ^26^, ^27^, ^28^ The DNA binding site of C.Csp231I consists of two sets of palindromic sequences (operators); however, unusually, there is a large (~ 18 bp) separation between them.^28^ C.Csp231I has the recognition sequence CTAAGN_5_CTTAG, where the inverted repeat sequences are separated by A-rich pentanucleotides (GAAAA and AAAAT, respectively, for the distal and proximal operators). The sequence between the two operator sites is also notably rich in polyA and polyT tracts, the significance of which is unknown but suggests a structural constraint on the DNA (Fig. 1a).

The C.Csp231I controller protein (*M*_r_ = 11,360) is significantly larger than those whose structures have been investigated to date (typically being 8000–9000). Comparison of the 98-amino-acid sequence of C.Csp231I with C.AhdI shows only a 29% identity over 62 core residues, with C.Csp231I having a 32-amino-acid extension at the C-terminus that is predicted to form two additional helices.^28^ By comparison, C.Csp231I and C.EcoO109I share an almost 70% sequence identity over the first 80 amino acid residues, consistent with the similarity of their DNA recognition sites (Fig. 1b).

In order to further our understanding of this group of transcriptional regulators, we embarked on the structural and functional analysis of this new class of C-proteins. Here, we present the X-ray crystal structure and solution studies of C.Csp231I.

## Results and Discussion

### DNA–protein and protein–protein interactions

**Fig. 2:**
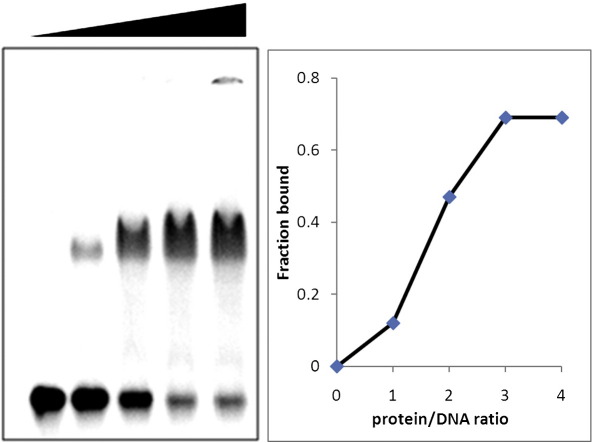
EMSA showing the interaction of C.Csp231I with a 25-bp DNA duplex containing the recognition sequence O_L_. The DNA concentration was 240 nM, and the protein/DNA ratios were 0, 1, 2, 3, and 4 (protein subunits per DNA duplex). The DNA duplex was formed from TCACACTAAGGAAAACTTAGTAAAA and its complementary strand.

The protein C.Csp231I was expressed and purified to homogeneity, as previously described.^28^ To confirm the DNA binding ability of the protein, we undertook electrophoretic mobility shift assays (EMSAs) using a 25-bp DNA sequence containing the recognition sequence (Fig. 2). The protein dimer binds with high affinity to this sequence. The sigmoidal nature of the binding curve suggests that binding at these submicromolar concentrations might be dominated by the monomer–dimer equilibrium of the C-protein. Indeed, the monomer–dimer equilibrium at a low concentration of C-protein is believed to be an important component of the genetic switch mechanism of other C-proteins, such that DNA binding (and thus transcription of the endonuclease) is delayed until there is a sufficient concentration of C-protein to generate active dimers.^19^

**Fig. 3:**
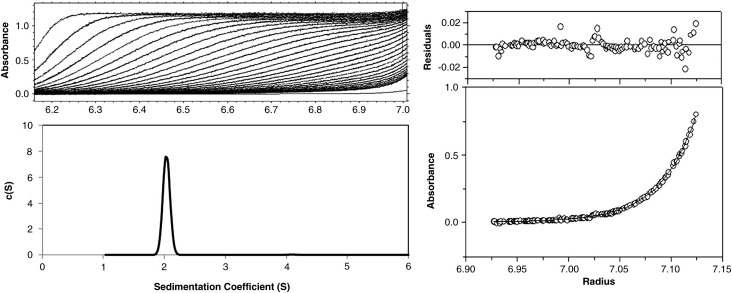
AUC of C.Csp231I. Top left: Sedimentation velocity: example of data fitted from a run at 40,000 rpm and scanning at 280 nm. Bottom left: Normalised *c*(*S*) distribution plots for a protein concentration of 80 μM. Right: Sedimentation equilibrium scan at 230 nm, with data fitted to a single-species model (*M*_r _= 22,300 ± 400) using a global fit to two concentrations (5 and 10 μM) at three speeds (20, 30, and 40 krpm). The fit is shown for data collected at a concentration of 5 μm and a speed of 40 krpm.

To further characterise hydrodynamic properties, we performed sedimentation velocity analytical ultracentrifugation (AUC) experiments at a range of protein concentrations (10–80 μM). Excellent fits to these data were obtained with tight residuals (Fig. 3). A clear single species was apparent in each *c*(*S*) plot, indicating a sedimentation coefficient of 2.0 S and a molecular weight of 22,000–23,000, corresponding closely to the predicted molecular weight of a dimer (Table 1). Sedimentation equilibrium studies confirmed the dimeric nature of the protein, and the curves fitted well to a single-species model (*M*_r_ = 22,300 ± 400) with a global fit. There was no concentration dependence observed and no evidence of monomers at the lowest measurable concentration (5 μM), consistent with a relatively low (submicromolar) *K*_d_ for dimerisation, as indicated by the DNA binding studies mentioned above.

**Table 1:** Hydrodynamic parameters of C.Csp231I

	Concentration (µM)	*S*	*f*/*f*_0_	*M*_r_	*R*_g_ (Å)	*D*_max_ (Å)
AUC	10	2.01^a^	1.30	22,600^b^		
	20	2.06^a^	1.33	23,100^b^		
	80	2.02^a^	1.34	22,900^b^		
SAXS	100			22,000^c^	20.7	62.0
HYDROPRO		2.09		22,700^d^	19.0	61.1

a Experimental sedimentation coefficient.

b Experimental *M*_r_ from AUC.

c Experimental *M*_r_ from the Kratky plot.

d Theoretical *M*_r_ from sequence.

### X-ray crystallographic analysis

The protein crystallised in two space groups: a monoclinic form (*P*2_1_) and a cubic form (*F*4_1_32).  Data collected from both crystal forms at the Diamond Light Source (UK) extended to around 2.0 Å with good statistics (Table 2). The calculated Matthews coefficients were 2.38 Å^3^ Da^− 1^ (one monomer in the asymmetric unit) and 2.01 Å^3^ Da^− 1^ (two monomers in the asymmetric unit) for the cubic and monoclinic structures, respectively.^29^ Thus, there is a crystallographic dyad between the two subunits in the cubic form, whereas the subunits are related by a noncrystallographic dyad in the monoclinic form. Molecular replacement resulted in strong solutions, and clear difference density was observed for the extended helical regions that were absent in the search model. Both structures refined well, with 99% of the residues lying in the preferred regions of the Ramachandran plots, and the *R*_work_/*R*_free_ values and bond geometries were reasonable for the resolution cutoff of 2.0 Å (Table 1). The final electron density maps were of high quality (Fig. S1), with only the two C-terminal residues (out of 98) absent from each model.

**Table 2:** Crystal, data collection, and refinement parameters

Crystal parameters	Cubic form	Monoclinic form
Space group	*F*4_1_32	*P*2_1_
Cell dimensions		
*a*, *b*, *c* (Å)	137.37, 137.37, 137.37	49.01, 29.53, 64.38
α, β, γ (°)	90.00, 90.00, 90.00	90.00, 101.91, 90.00
Solvent content (%)	48.3	38.7
Molecules in asymmetric unit	1	2

*Data collection*		
Wavelength (Å)	0.9795	0.9795
Resolution (Å)	19.2–2.0	50–2.0
Number of measured reflections	308,483	41,601
Number of unique reflections	7753	11,784
Completeness (%)	97.2 (94.2)	99.1 (99.8)
Mosaicity (°)	0.2	1.1
〈*I*/σ(*I*)〉	43.92 (12.02)	11.7 (4.1)
Multiplicity	39.8 (41.2)	3.3 (3.4)
*R*_merge_^a^	7.2 (40.8)	5.7 (27.69)

*Refinement parameters*		
*R*_work_/*R*_free_	17.8/22.5	20.7/24.6
Number of atoms/*B*-factors		
Protein	782/41.6	1568/33.3
Water	66/35.4	88/35.4
RMSD		
Bond lengths (Å)	0.023	0.009
Bond angles (°)	1.753	1.061

Values in parentheses are for the highest-resolution shell (2.11–2.00 Å).

a *R*_merge_ = ∑_*hkl*_∑_*i*_⏐*I*_*i*_(*hkl*) − 〈*I*(*hkl*)〉⏐∑_*hkl*_∑*_i_I_i_*(*hkl*), where 〈*I*(*hkl*)〉 is the mean intensity of reflection *I*(*hkl*), and *I*_*i*_(*hkl*) is the intensity of an individual measurement of reflection *I*(*hkl*).

**Fig. 4:**
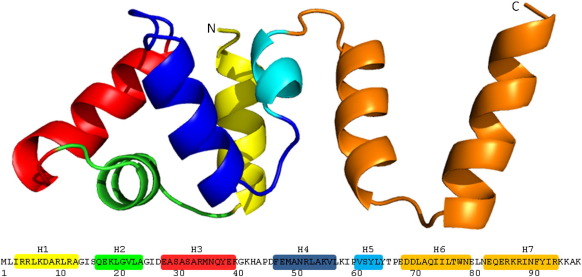
Topology of the monomer. A cartoon of a single monomer of C.Csp231I is shown with the corresponding amino acid sequence. All 98 amino acids are visible in the X-ray structure. Helices 1–5 are highly conserved within the C-protein family and form a tight globular domain. C-terminal helices 6 and 7 represent an additional domain not observed in any other C-proteins.

Previous sequence alignments indicated that C.Csp231I has a large C-terminal extension compared with other C-proteins.^28^ The predicted α-helical nature of this extension is confirmed by the crystal structures presented here, where the overall topology comprises a compact five-helix bundle with two extended C-terminal helices (Fig. 4). The N-terminal core retains a fold common to other known C-protein structures, such as C.AhdI,^18^ C.BclI,^21^ and C.Esp1396I,^22^ and is closely related to other transcriptional regulatory proteins such as SinR.^30^ However, the two additional C-terminal helices (Fig. 4, orange) provide a new scaffold that is distal from the helix–turn–helix motifs (Fig. 4, green and red).

### Structure of the protein dimer

**Fig. 5:**
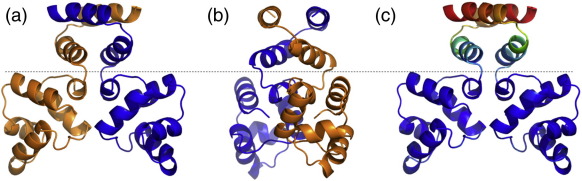
Biological unit of C.Csp231I. (a and b) A cartoon of the C.Csp231I dimer in two orientations. The region below the broken line corresponds to the typical C-protein family structure composed of a 10-helix dimer. The region above this line represents the additional domain formed by the two C-terminal helices from each monomer. (c) In order to highlight mobile regions, we have coloured the dimer according to C^α^*B*-factor values calculated following refinement using a scale between 18 (dark blue) and 112 (dark red).

Structures from the two alternative space groups are very similar, and a least-squares fit of the monomer from the cubic unit cell data to either of the monomers from the monoclinic unit cell data gave an RMSD of 0.94 Å. A comparison of the dimers (formed by a crystallographic dyad in the cubic form and by a noncrystallographic dyad in the monoclinic form) resulted in an RMSD of 1.31 Å, reflecting a slight hinge movement at the dyad interface. Analysis of the crystal packing from both structures reveals a strong dimer interface that is extended compared to other C-proteins due to the interaction between the additional C-terminal helices (Fig. 5a and b). The following analysis is based on the cubic form of C.Csp231I. The total dimer interface area of C.Csp231I is 1410 Å^2^ compared to 1040 Å^2^ in C.Esp1396I and 710 Å^2^ in C.AhdI. The striking feature in C.Csp231I is not that the dimer interface is larger, but rather that 75% of the dimer interface is contributed by the extended C-terminal helical region (helices 6 and 7; residues 67–98). This is achieved by the interleaving of the final two helices from each monomer. In fact, the five-helix bundle that makes up the main conserved domain only creates 310 Å^2^ of buried surface area on its own. Surprisingly, this domain contains the only pair of interchain H-bonds (Ser62-Thr66); in contrast, C.AhdI and C.Esp1396I contain a total of four and five interchain H-bonds, respectively. Moreover, in C.Csp231I, there are more nonbonded (van der Waals) intersubunit contacts between helices 6 and 7 than there are between the major domains of the two subunits (57 compared to 50). Overall, this results in a very different dimer interface compared to those C-protein structures solved to date. A similar analysis of the monoclinic form of C.Csp231I reveals the same major features at the dimer interface, with an equivalent pair of interchain H-bonds. The buried surface area is reduced by ca 200 Å due to a small outward rotation of the final C-terminal helices relative to each other (residues 88–96), which may in part be due to the observed differences in crystal packing contacts in this region.

**Fig. 6:**
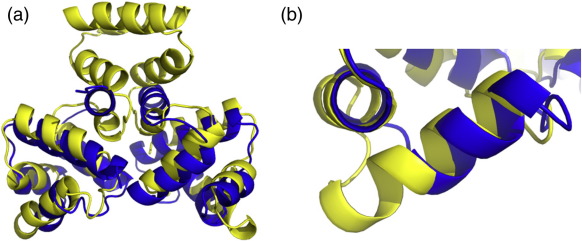
Comparison to structural homologoues. (a) A structural superposition of C.Csp231I (yellow) and C.Esp1396I (blue). C.Csp231I shows an overall expansion of the HTH domains and a compression of helix 5 within the main globular region of the dimer. (b) HTH region, aligned on the scaffold helix 2, demonstrating the extension of the major groove recognition helix 3 in C.Csp231I.

From the X-ray structures, the overall dimensions of the C.Csp231I dimer are 50 Å ×  30 Å ×  50 Å, compared with the more typical size of C.Esp1396I at 50 Å × 30 Å × 25 Å. In common with the other C-proteins, there is a strong hydrophobic core that accounts for the stability and observed low *B*-factors of the globular region. In addition to the unusual dimer interface, it is notable that the recognition helix of the HTH motif is extended by one turn compared to other C-proteins (Fig. 6). This might be accounted for by the larger (5–6 bp) inverse repeats found in the motif 8 class of controller proteins.

### Dynamics and flexibility

The analysis of *B*-factors in the cubic crystal form of C.Csp231I reveals that the extended C-terminal region of the protein, comprising helices 6 and 7, is significantly more mobile than the rest of the structure (Fig. 5c). This is not so apparent in the monoclinic form, where crystal packing forces stabilise this region and the *B*-factors are more evenly distributed throughout the entire structure. It is therefore possible that the extended C-terminal helical region, particularly helix 7, may be mobile in solution in the absence of stabilising interactions. It is not clear at this stage how this region influences biological function, but presumably it could have a role in DNA and/or protein–protein interactions, potentially becoming more rigid following binding.

We were interested to know the behaviour of these potentially flexible regions in the solution environment. The program HYDROPRO^31^ was used to calculate theoretical hydrodynamic parameters based on the C.Csp231I dimer structure (Table 1). The resulting theoretical sedimentation coefficient of 2.1 S is in very close agreement with the calculated value of 2.0 S, suggesting that the crystal structure resembles the structure in solution.

**Fig. 7:**
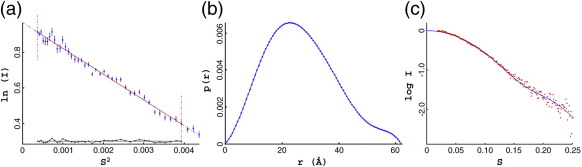
SAXS. (a) A Guinier plot of the scattering curve is shown with a line of best fit and residuals. (b) Plot of the *p*(*r*) function. (c) Theoretical scattering curve generated from the C.Csp231I dimer structure (blue) plotted against the observed scattering data (red points).

More detailed studies by small-angle X-ray scattering (SAXS) were then performed on the free protein in solution (Fig. 7). The resulting analysis gave an *R*_g_ of 20.7 Å and a *D*_max_ of 62 Å, consistent with the majority of the protein being in a folded globular state, including the additional C-terminal helices (see Table 1). Moreover, the *M*_r_ of the protein obtained from the Kratky plot (22,000) matches that expected for a protein dimer (22,700). Finally, a comparison of the theoretical scattering curve derived from the X-ray coordinates with the experimental solution scattering curve reveals an excellent fit (Fig. 7c). The globular structure of the free protein, as seen in both cubic and monoclinic crystals, is therefore representative of the structure in solution, at least under the buffer conditions tested.

### The DNA binding surface

**Fig. 8:**
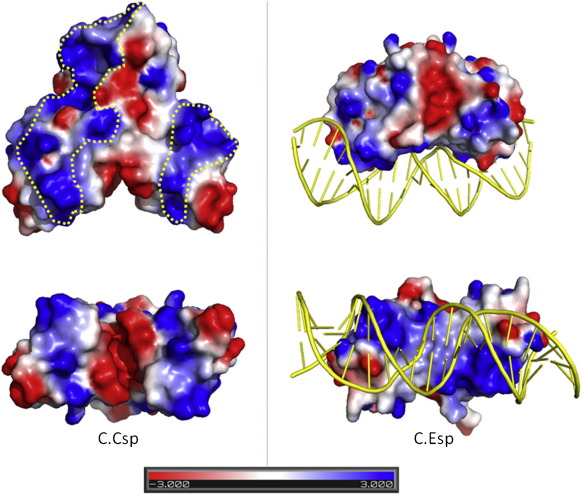
Charge distribution. A comparison of the electrostatic surface of C.Csp231I (left column) and a representative portion of the C.Esp1396I protein–DNA complex structure (Protein Data Bank ID: 3CLC). The lower views are orientated 90º around the horizontal axis from the upper views to expose the HTH regions. The electrostatic potential is rendered on the surface of the proteins using a colour scale between − 3.0*kT* e^− 1^ (red) and 3.0*kT* e^− 1^ (blue).

In order to predict potential DNA binding regions, we mapped the electrostatic surface potential for comparison with other C-proteins (Fig. 8) using a region of the C.Esp1396I nucleoprotein complex structure (Protein Data Bank ID: 3CLC). The overall charge distributions between the other available C-protein structures are similar, with a flat base of positive charge at the DNA binding interface. It is also common to see an extension of this positively charged region from the recognition helices, around the surface towards the scaffolding helices. In the C.Esp1396I DNA structure, these positive patches can be seen to aid in DNA bending, as interacting phosphates are wrapped around the base of the protein. Between C-proteins, the remaining surface charges are fairly evenly distributed, with the exception of occasional small patches of negative charge. However, the electrostatic surface of C.Csp231I is strikingly different: there is a strong region of negative charge located between the two recognition helices at the predicted DNA binding surface, and the overall charge distribution is highly polarised. In C.Esp1396I, the DNA can be seen to lie across a flat surface consisting of positive/neutral/positive patches. Moreover, the corresponding region in C.Csp231I does not form a flat base, but rather a V-shaped cleft with a strong negative patch in the centre, potentially some distance from the bound DNA.

The other notable difference from other C-proteins is the presence of an almost continuous band of positive charge that runs around the C.Csp231I dimer (highlighted in Fig. 8). This narrow region extends from the classical DNA binding interface between the HTH recognition helices towards and over the extended C-terminal region, and is mirrored on the opposite side as a result of the dyad symmetry. It may be that these extended positive surfaces, having the potential to loop the DNA around the entire structure, can make additional contacts with DNA.

**Fig. 9:**
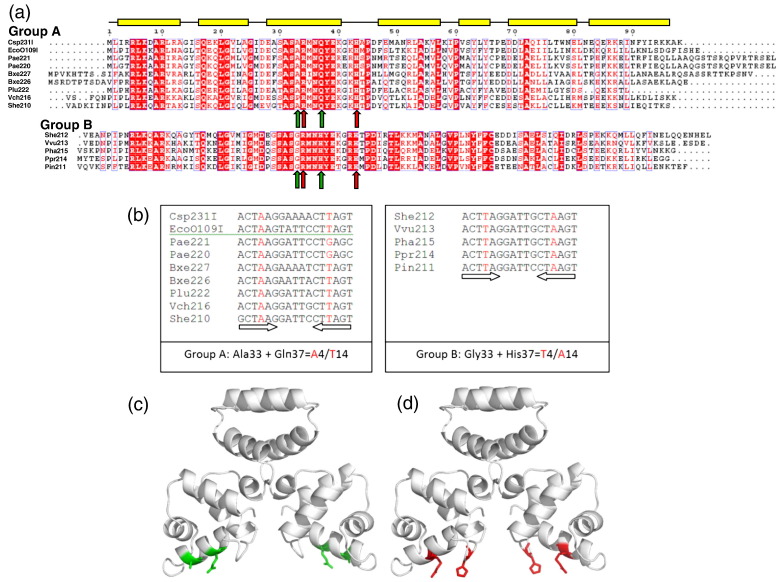
Conservation within the motif 8 group. The motif 8 controller proteins can be divided into two classes based on the conservation of amino acids in the putative DNA binding region and their corresponding recognition sites. (a) Groups A and B are shown (top and bottom, respectively) with identical residues in red boxes and similar residues in red text. The secondary structure of C.Csp231I is shown above, where yellow boxes represent the seven helices as in Fig. 4. Arg34 and His43 (invariant) are highlighted with red arrows. Ala33 + Gln37 (group A only) and Gly33 + His37 (group B only) are highlighted with green arrows. (b) The recognition sequences for each group are shown with bases specific for the group in red text. The positions of the inverted repeats are depicted with arrows. (c) Model showing the positions of residues Ala33 and Gln37 in each monomer (green) and of (d) residues Arg34 and His43 (red).

An alignment of the 14 motif 8 protein sequences identified in C-proteins by Sorokin *et al.* reveals several highly conserved residues that can be mapped onto the putative DNA binding region of C.Csp231I.^14^ In fact, the most conserved region is found in the recognition helix, with invariant residues such as Arg34 and His43 facing towards the solvent in an ideal position to make direct contacts with the DNA following binding. Other residues in this region can be directly associated with their putative DNA recognition sites. For example, we have divided the motif 8 group into two subgroups based on the amino acids at positions 33 and 37. Group A, with Gln37 invariant (and Ala33 almost so), has mainly A4/T14 bases in the inverted repeats, while group B, with His37 invariant (and Gly33 almost so), has only T4/A14 in its recognition sequence (Fig. 9). It is therefore likely that Gln37 and Ala33 in C.Csp231I make direct contacts with these bases.

## Materials and Methods

### Expression, purification, and crystallisation

The full details of cloning and purification are given by Streeter *et al.*^28^ Briefly, the native untagged C.Csp231I protein was overexpressed in *Escherichia coli* BL21(DE3) Gold cells from a pET-11a plasmid containing the *csp231IC* gene (GenBank ID: AY787793.1). The protein was purified with a three-step column chromatography method using an AKTA purifier and the following columns (GE Healthcare): HiTrap heparin, HiTrap SP, and, finally, 26/60 Sephacryl S-100 HR. Crystallisation was performed by employing the hanging-drop vapour-diffusion method using the PACT screen kit (Molecular Dimensions). The purified protein (1.2 mg ml^−^^ 1^) was mixed at a 1:1 ratio with the reservoir solutions (2 μl + 2 μl) and incubated at 289 K. Two conditions were found to yield strongly diffracting crystals: buffer 1 [0.1 M Na-Hepes (pH 7.5) and 1.4 M trisodium citrate dehydrate] produced a cubic form (*F*4_1_32), while buffer 2 [0.1 M malate–4-morpholineethanesulfonic acid–Tris (pH 7.0) and 20% polyethylene glycol 1500] produced a monoclinic form (*P*2_1_).

### X-ray crystallography and structure solution

Crystals were cryoprotected by transfer to a crystallisation solution containing 30% vol/vol glycerol prior to cryocooling in liquid nitrogen. Data were collected from two crystal forms at beamline IO2 at the Diamond Light Source. Crystals were maintained at 100 K using an Oxford Instruments Cryojet XL, and data were collected using an ADSC Q315 CCD detector. Oscillation widths of 0.5° for monoclinic data and 1.0° for cubic data were employed based on the unit cell parameters and mosaicity values (Table 2). Data were processed with either XDS and XSCALE,^32^ or MOSFLM^33^ and SCALA.^34^

Each structure was solved by molecular replacement with Phaser^35^ using a monomer of the C-protein C.Esp1396I as search model (Protein Data Bank ID: 3G5G^22^). From these initial phases, the additional extended regions were completed with reiterative rounds of building and refinement in Coot^36^ and REFMAC5.5,^37^ respectively. Stereochemical quality was analysed using PROCHECK,^34^ biological interfaces were analysed using PISA^38^ and PDBsum,^39^ and electrostatic surfaces were calculated with DELPHI.^40^, ^41^ All structural figures were produced using PyMOL (Schrödinger, LLC).

### Small-angle X-ray scattering

SAXS was carried out at the Diamond Light Source on beamline I22 equipped with a photon counter detector.^42^ Solutions of purified C.Csp231I at a concentration of 1.2 mg ml^− 1^ were loaded into mica-windowed cells that were temperature controlled to 16 °C. The beam was focused onto the detector placed at a distance of 2.25 m from the sample cell. The range of momentum transfer covered was 0.015 < **q** < 0.55 Å^− 1^, where **q** is the scattering vector (4πsinθ/λ) and λ = 1.0 Å is the X-ray wavelength. To check for radiation damage and aggregation during the SAXS experiment, we collected the data in 180 successive 1-s frames. The data were normalised to the intensity of the incident beam, and scattering of the buffer was subtracted using in-house programs. The averaged curves were processed using PRIMUS^43^ and GNOM^44^ to analyse the Guinier region and to generate the *p*(*r*) plot. CRYSOL^45^ was used to generate a theoretical scattering curve from the X-ray structure coordinates for comparison with the experimental data.

### Analytical ultracentrifugation

Sedimentation velocity experiments were performed in a Beckman XL-A analytical ultracentrifuge equipped with an An50-Ti rotor. Double-sector Epon cells with path lengths of 1.2 cm were used with quartz window assemblies. The final protein concentrations were in the range 10–80 μM in a buffer containing 50 mM Tris–HCl (pH 8.0), 100 mM NaCl, and 1 mM Na_2_-ethylenediaminetetraacetic acid. Samples were equilibrated at 20 °C and then accelerated to 40,000 rpm. Radial scans were performed at 10-min intervals at 280 nm. The partial specific volume for C.Csp231I was calculated from the amino acid composition using SEDNTERP^46^ at 0.745 ml g^− 1^, with a buffer density of 1.00397 g ml^− 1^ and a viscosity  of 0.01002 P. Analysis of the scans was performed using the program SEDFIT.^47^ Hydrodynamic parameters were calculated from the X-ray structure coordinates using the program HYDROPRO, version 7c.^31^

Sedimentation equilibrium experiments were performed in six channel cells with path lengths of 1.2 cm using  90-μl solutions of protein at concentrations of 5 and 10 μM. Corresponding cells were filled with 100 μl of sample buffer. The rotor was accelerated to speeds of 20, 30, and 40 krpm, and scans of absorbance at 230 nm *versus* radial displacement were taken at a resolution of 0.001 cm for times up to 21 h. The samples were maintained at a temperature of 20 °C. Analysis was performed with the ORIGIN software package (Beckman Coulter).

### DNA electrophoretic gel retardation assays

EMSAs were performed using nondenaturing gel electrophoresis. Complementary DNA strands corresponding to the 25-bp left operator upstream of the C.Csp1396I gene were purchased (Eurogentec), and the two strands were annealed to form a duplex (see Fig. 2 for sequences). Aliquots of C.Csp231I were incubated with 240 nM 5′ γ-^33^P-labelled DNA duplex in binding buffer (50 mM Tris–HCl, pH 8.0) at 4 °C for 30 min. The samples were loaded onto a prerun 8% native polyacrylamide gel and run in buffer containing 22 mM Tris base, 22 mM boric acid, and 0.5 mM ethylenediaminetetraacetic acid at 100 V. The gels were dried and then scanned using an FLA-5000 imaging system (FujiFilm).

### Sequence analysis

Amino acid and DNA sequence alignments were performed using ClustalW.^48^ The program ESPript^49^ was used to visualise the protein sequence alignments.

### Accession numbers

Coordinates and structure factor files have been deposited in the Protein Data Bank with accession codes 3LFP and 3LIS.

The following are the supplementary materials related to this article.

Fig. S1 Electron density. A region of the model is shown with the corresponding 2*F*_o _− *F*_c_ electron density at 2.5 σ (0.7 e^−^ Å^− 3^). Tyr65 residues are shown in the centre of the figure (one from each monomer) in blue and green.
