# Supplementary material for: Structural Analysis of a Novel Class of R–M Controller Proteins: C.Csp231I from Citrobacter sp. RFL231
Source: J Mol Biol. 2011 Jun 3;409(2-4):177–88. doi: 10.1016/j.jmb.2011.03.033 (PMC3115060; doi:10.1016/j.jmb.2011.03.033)
Supplement: Fig. S1 — Electron density. A region of the model is shown with the corresponding 2Fo − Fc electron density at 2.5 σ (0.7 e− Å− 3). Tyr65 residues are shown in the centre of the figure (one from each monomer) in blue and green. [file mmc1.doc]

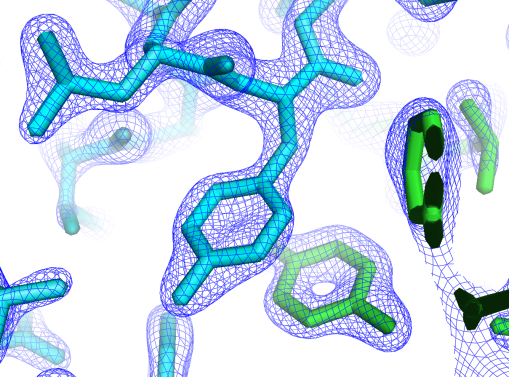


**Supp Fig 1. Electron density.** A region of the model is shown with corresponding 2*F*_o_-*F*_c_ electron density at 2.5σ (0.7 e^-^/Å^3^). Residues Tyr65 are shown in the centre the picture, one from each monomer in blue and green.
